# A New Fuzzy-Evidential Controller for Stabilization of the Planar Inverted Pendulum System

**DOI:** 10.1371/journal.pone.0160416

**Published:** 2016-08-02

**Authors:** Yongchuan Tang, Deyun Zhou, Wen Jiang

**Affiliations:** School of Electronics and Information, Northwestern Polytechnical University, Xi’an, Shaanxi 710072, P.R. China; Southwest University, CHINA

## Abstract

In order to realize the stability control of the planar inverted pendulum system, which is a typical multi-variable and strong coupling system, a new fuzzy-evidential controller based on fuzzy inference and evidential reasoning is proposed. Firstly, for each axis, a fuzzy nine-point controller for the rod and a fuzzy nine-point controller for the cart are designed. Then, in order to coordinate these two controllers of each axis, a fuzzy-evidential coordinator is proposed. In this new fuzzy-evidential controller, the empirical knowledge for stabilization of the planar inverted pendulum system is expressed by fuzzy rules, while the coordinator of different control variables in each axis is built incorporated with the dynamic basic probability assignment (BPA) in the frame of fuzzy inference. The fuzzy-evidential coordinator makes the output of the control variable smoother, and the control effect of the new controller is better compared with some other work. The experiment in MATLAB shows the effectiveness and merit of the proposed method.

## 1 Introduction

The planar inverted pendulum system [1, 2] was generalized from the linear inverted pendulum [3, 4]. Both of them are self-unstable, high-order, multi-variable and nonlinear system, while the planar inverted pendulum system is more sophisticated than the linear inverted pendulum because of a higher degree of freedom and a stronger coupling in both axes. The detailed mathematical model and the introduction to the physical structure of the planar inverted pendulum are presented in [5]. The stability of the planar inverted pendulum system in the sense of Lyapunov is proved in [6] based on the mathematical model of the system. The planar inverted pendulum system is an effective benchmark to verify the effectiveness of a control method. Researches on stabilization of the planar inverted pendulum system are of great help to some key technologies in rocket, missile and robotics control methods, for example, the equilibrium of a humanoid robot for its bipedal walking [7]. Some researches on the planar inverted pendulum have been reported in recent years based on various methods including the linear quadratic regulator (LQR) method [8, 9], the neural network control theory [1], the sliding mode control theory [5, 10] and the fuzzy control theory [2, 11, 12].

Fuzzy control theory [13, 14] is based on fuzzy sets [15] and fuzzy inference [16, 17]. So far, fuzzy inference has been extensively used in real applications including risk analysis [18–22], controller design [23–25], decision-making [26, 27], and so on [28–30]. Similar to fuzzy sets theory, Dempster-Shafer theory of evidence or evidence theory [31, 32] is effective in uncertain representation and data fusion [33, 34]. Evidential reasoning [35–37] is effective in dealing with problems related to decision-making [38, 39], pattern recognition [40, 41], fault diagnosis and risk analysis [42, 43], human reliability analysis [44], environment protection [45], and so on [46–48]. Both fuzzy inference and evidential reasoning are effective ways for empirical knowledge representation and process. There are already some studies incorporate fuzzy sets theory with evidence theory for controller design [23, 49, 50], classification [51], knowledge management [52], fault diagnosis [53], and so on [54]. In [23], Yager and Filev introduce evidence theory into the basic fuzzy system model to handle the probabilistic uncertainty of the consequent in fuzzy rules. With the combination of evidential reasoning and fuzzy inference, Graham [49] fuses the sensory information of the robot’s environment to fulfill the collision avoidance control of robots. An expert system is designed with a fuzzy logic controller in [50], while the output of the expert system for decision making is suggested with a belief level accomplished by evidential reasoning.

The existing researches show that a hybrid intelligent approach can make each conventional intelligent algorithm more effective and flexible for dealing with the problems in real applications [52–56]. In this paper, a fuzzy-evidential controller for the planar inverted pendulum system is proposed. This work not only addresses the uncertainty in the consequent of fuzzy rules as it is in [23], but also coordinates the coupling from different fuzzy controllers with the proposed fuzzy-evidential coordinator. The empirical knowledge for stabilization of the planar inverted pendulum system is expressed by fuzzy rules while designing the fuzzy nine-point controller, and a coordinator for different control variables is designed based on the dynamic basic probability assignment (BPA) in the frame of fuzzy inference. The control strategy in the proposed controller doesn’t depend on the mathematical model of the controlled system, which means a shorter developing period and lower cost in real application. The experiment in MATLAB shows that the proposed method is effective. In addition, the proposed fuzzy-evidential controller forces the planar inverted pendulum into its equilibrium state faster than the fuzzy controller [12] and the LQR method [8].

The rest of this paper is organized as follows. In Section 2, some preliminaries are briefly introduced. In Section 3, a fuzzy-evidential controller for the planar inverted pendulum system is presented. In Section 4, the control experiment in MATLAB verifies the effectiveness and the merit of the proposed method. The conclusions are given in Section 5.

## 2 Preliminaries

Some basic concepts of fuzzy control theory and evidence theory are presented in this section, as well as an introduction to the planar inverted pendulum system.

### 2.1 Fuzzy control theory

The fuzzy control theory [13, 14] is based on the fuzzy sets [15] and fuzzy inference [16, 17]. Some key steps of designing a fuzzy controller are shown as follows [13, 14].

- *Step 1*. Defining the structure of the fuzzy controller.
- *Step 2*. Defining the input and output fuzzy sets.
- *Step 3*. Defining the membership function of input and output function.
- *Step 4*. Defining the fuzzy control rules and the fuzzy inference process.
- *Step 5*. Solving the fuzziness with defuzzifier.

A *fuzzy* *set* [13–15] *A* in *X* is a set of ordered pairs, denoted as *A* = {(*x*, *u*_*A*_ (*x*))}, where *X* denotes a collection of objects (points) denoted by *x*, and *u*_*A*_(*x*) is the *membership* *grade* of *x* in *A*, *u*_*A*_: *X* → *M* is a function from *X* to the *membership* *space* *M*.

The equation of *singleton* *fuzzifier* is shown as follows [57, 58]:

$$u_{A}\left(x\right)=\left\{\begin{array}{c} 1,x=x^{*}\\ 0,\text{others} \end{array},\right.$$ (1)

where the point x* ∈ *X* is mapped as a singleton in *X*, and its grade of membership is 1, while the others are 0. The equation of *weighted* *average* *defuzzifier* is shown as follows [57, 58]:

$$v^{*}=\frac{\sum_{i=1}^{M}v_{i}\cdot w_{i}}{\sum_{i=1}^{M}w_{i}},$$ (2)

where *v*_*i*_ ∈ *V*, and *V* is the output space of a fuzzy controller, *M* is the *membership* *space*, *w*_*i*_ is a ratio corresponding to the response characteristic of a system.

### 2.2 Dempster-Shafer theory of evidence

In this section, some basic concepts of the evidence theory [31, 32] are introduced, including the frame of discernment, basic probability assignment (BPA) and Dempster’s rule of combination.

#### Frame of discernment

Evidence theory assumes a finite nonempty set of mutually exclusive events Θ = {*θ*_1_,*θ*_2_, …,*θ*_n_}, a power set 2^|Θ|^ is defined as the frame of discernment, shown as follows [31, 32]:

$$2^{\left|\Theta \right|}=\left\{\emptyset ,\left\{\theta_{1}\right\},\left\{\theta_{2}\right\},\ldots ,\left\{\theta_{n}\right\},\left\{\theta_{1},\theta_{2}\right\},\ldots ,\left\{\theta_{1},\theta_{2}\ldots ,\theta_{n}\right\}\right\}.$$ (3)

#### Basic probability assignment

The basic probability assignment(BPA) function or mass function *m* is defined as a mapping from the power set of Θ to a number between 0 and 1, which satisfies [31, 32]:

$$m\left(\emptyset \right)=0,0\leq m\left(A\right)\leq 1,\sum_{A\in \Theta }m\left(A\right)=1,$$ (4)

where ∅ is an empty set, *A* is any subsets of Θ, the mass function *m*(*A*) represents how strongly the evidence supports *A*. The mass *m*(Θ) represents the uncertainty of the evidence.

#### Dempster’s rule of combination

Dempster’s rule of combination combines two BPAs in a way that the new BPA represents a consensus of the contributing pieces of evidence, it sets intersection putting the emphasis on the common elements of evidence. Dempster’s rule of combination is the orthogonal sum of *m*_1_ and *m*_2_, denoted by (*m*_1_ ⊕ *m*_2_), shown as follows [31, 32]:

$$m\left(A\right)=\left(m_{1}⊕m_{2}\right)\left(A\right)\;=\frac{1}{1-k}\sum_{B\cap C=A}m_{1}\left(B\right)\cdot m_{2}\left(C\right),$$ (5)

where *A*, *B*, and *C* are subsets of 2^|Θ|^, *k* is a normalization constant representing the conflict coefficient of two BPAs, *k* is defined as follows [31, 32]:

$$k=\sum_{B\cap C=\emptyset }m_{1}\left(B\right)\cdot m_{2}\left(C\right).$$ (6)

#### Pignistic probability transformation

Pignistic probability transformation *BetP*(⋅) in the transferable belief model (TBM) [59] is commonly chosen to transform a BPA to probability distribution. *BetP*(*A*) is defined as follows:

$$BetP\left(A\right)=\sum_{B\in 2^{\theta },A\subseteq B}\frac{\left|A\cap B\right|}{B}m\left(B\right),$$ (7)

where *A* ∈ 2^*θ*^, |*A*| is the cardinality of subset *A*.

For detailed information related to evidence theory, one can refer [31, 32, 46, 59].

### 2.3 The planar inverted pendulum system

**Fig 1:**
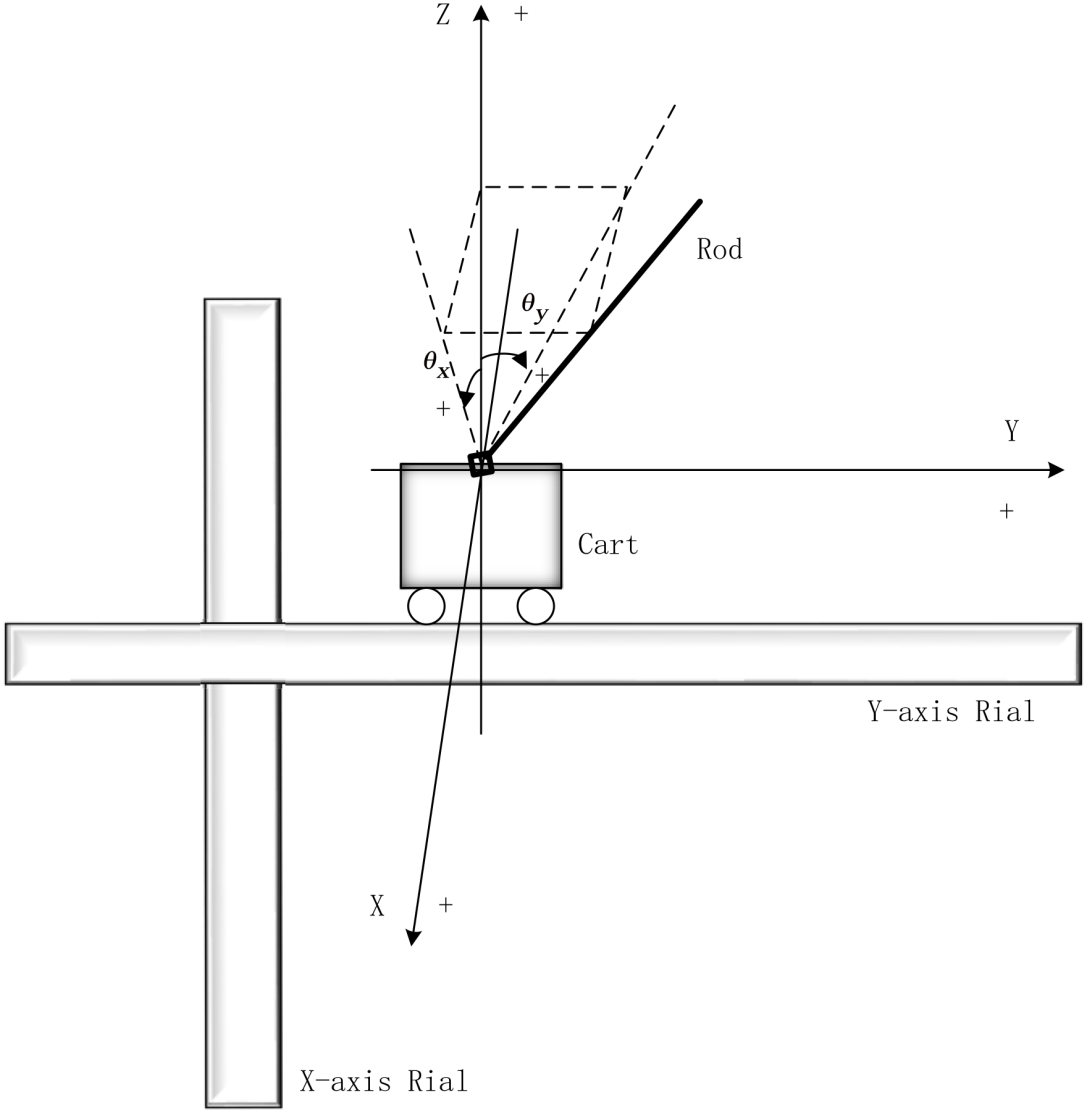
The setup of the planar inverted pendulum system.

The planar inverted pendulum system [1, 2] consists of a rod, a cart and two rails in the orthogonal axes, as is shown in Fig 1. The cart can move along the rail in *Y* axis, and the rail in *Y* axis can move along the rail in *X* axis, so the cart can move within the planar plane *XOY*. The rod is set on the cart by a Hooke’s joint, it can rotate around *X* axis and *Y* axis. Normally, the pendulum deviates from *OZ* direction with *θ*_*x*_ in *XOZ* plane, and *θ*_*y*_ in *YOZ* plane. Fig 1 also shows the positive direction for each axis and each angle with the plus symbol (+), so the unmarked direction is the negative direction. The stabilization of the planar inverted pendulum system includes the equilibrium of the rod accomplished by the pendulum controller and controlling the cart back to the origin of coordinates by the cart controller in both *X*-axis and *Y*-axis.

According to the control logic, if the pendulum deviates from the upright position clockwise (Clockwise is chosen as the positive direction), the pendulum controller should control the cart move toward the right direction (Defining on the left is the positive direction), in this way, the pendulum will go back toward its equilibrium position by rotating anticlockwise. On the contrary, if the pendulum deviates from the upright position anticlockwise, the pendulum controller should control the cart move toward the left direction, and the pendulum will go back to its equilibrium position by rotating clockwise.

The cart controller will control the cart indirectly, this is because the equilibrium of the rod is the foundation of the stabilization of the whole system, so controlling the cart back to the origin of coordinates has a lower priority level than the rod’s equilibrium. If the cart leaves the original point toward the positive direction, the cart controller should keep its movement to the positive direction, this behavior will lead the pendulum deviate from the upright position anticlockwise, so the pendulum controller will control the cart move toward the negative direction, thus the pendulum will rotate clockwise and go back to its equilibrium position, also, the cart will go back to its original position automatically. If the cart leaves the original point toward the negative direction, the control logic will be on the contrary.

More detailed information about the mathematical model and the physical structure of the planar inverted pendulum is presented in [1, 2, 5, 8], and the stability of the system in the sense of Lyapunov is proved in [6].

## 3 Fuzzy-evidential controller

For each axis of the planar inverted pendulum system, the control mode is assumed to be the same, which means the controller for each axis is designed with the same way. The control force for each axis comes from a single motor, however, according to the control logic analyzed above, there should be two controllers in each axis, a controller for the rod and a controller for the cart. Thus, in each axis, after designing a fuzzy controller for the rod and another one for the cart, respectively, a coordinator for these two controllers is necessary to output the final control force of the motor, denoted as *F*. The function of the control force *F*, which is similar to the adaptive sliding-mode control in [5], is shown as follows:

$$F=m_{1}\cdot F_{1}+m_{2}\cdot F_{2},$$ (8)

where *m*_1_ and *m*_2_ are the output of the designed coordinator based on fuzzy inference and evidential reasoning, which will be shown in detail in section 3.2. The *F*_1_ and *F*_2_ are the output of the fuzzy nine-point controller of the rod and the cart, which will be shown in detail in section 3.1.

**Fig 2:**
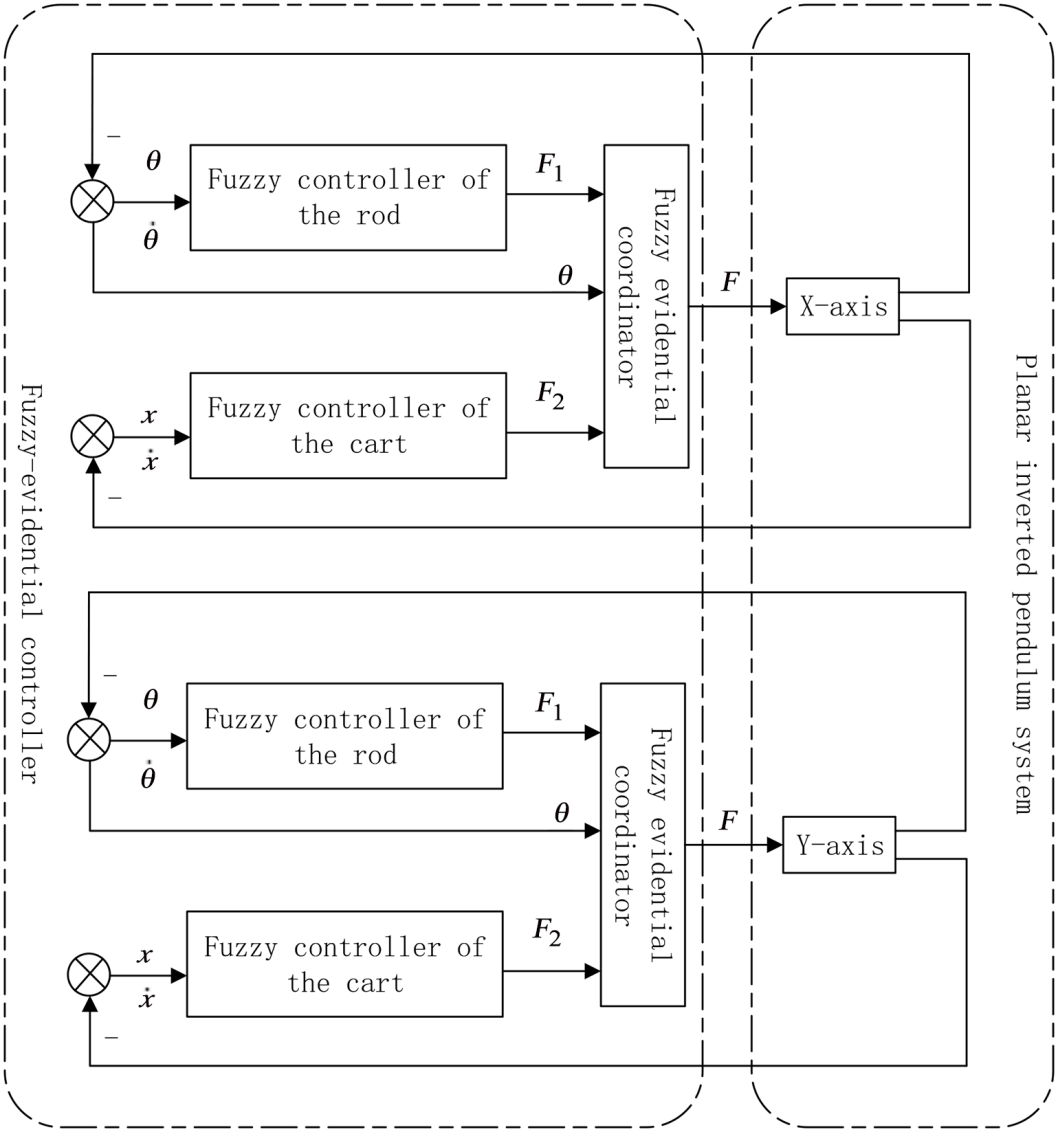
Fuzzy-evidential controller for the planar inverted pendulum system.

The proposed fuzzy-evidential controller for the planar inverted pendulum system is shown in Fig 2. In Fig 2, *θ* is the angular deviation of the rod, and its derivative $\dot{\theta }$ means the angular speed. *x* is the displacement deviation of the cart, and its derivative $\dot{x}$ means the speed of the cart.

### 3.1 Fuzzy controller of the rod and cart

**Fig 3:**
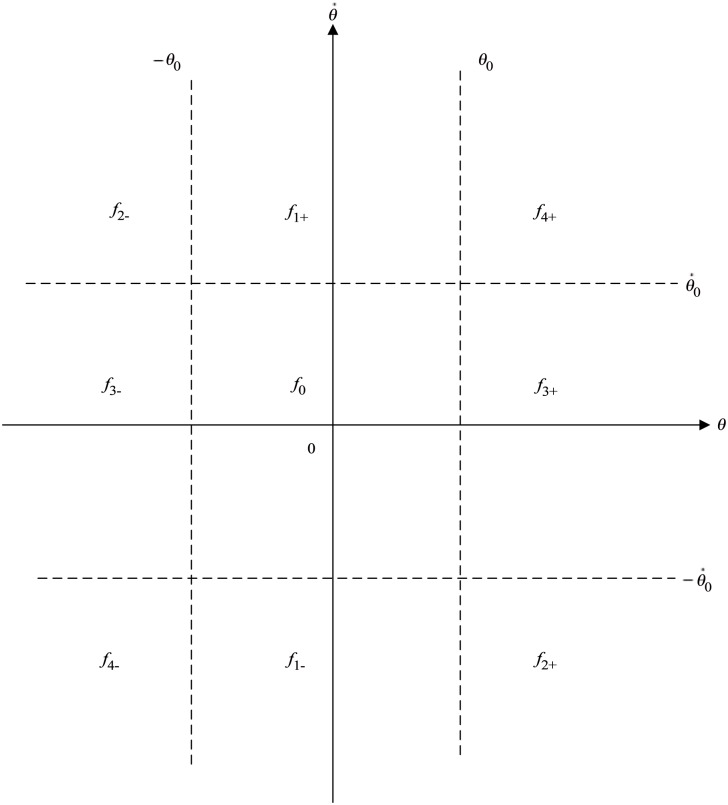
Principles of the nine-point controller.

Multi-point controller is a kind of direct and simple control strategy based on control experience [60], and a nine-point controller [61] can be regarded as a special case of the multi-point controller. In this paper, the fuzzy nine-point controller is chosen to be the fuzzy controller for the rod and the cart in Fig 2. Take the control variable *θ* for example, the principle of the nine-point controller is shown as Fig 3.

In Fig 3, a phase plane consists of the control variable *θ* and its gradient $\dot{\theta }$ is divided into nine parts by the zero zone value *θ*_0_, −*θ*_0_, ${\dot{\theta }}_{0}$ and $-{\dot{\theta }}_{0}$. There are nine principles in fuzzy nine-point controller [61]. If the system state is *θ* > *θ*_0_ and $\dot{\theta }>{\dot{\theta }}_{0}$, which means the angular deviation of the rod is very big in positive direction, so the system needs the strongest control effect *f*_4+_ in positive direction to enlarge the output of the rod’s fuzzy controller and reduce the deviation of the rod. The other eight control zones can be under control in a similar way, the rest eight principles are shown as follows.

- If the system state is *θ* > *θ*_0_ and $|\dot{\theta }|\leq {\dot{\theta }}_{0}$, applying a strong control effect *f*_3+_ in positive direction to the system.
- If the system state is *θ* > *θ*_0_ and $\dot{\theta }<-{\dot{\theta }}_{0}$, applying a weak control effect *f*_2+_ in positive direction to the system.
- If the system state is |*θ*| ≤ *θ*_0_ and $\dot{\theta }>{\dot{\theta }}_{0}$, applying the weakest control effect *f*_1+_ in positive direction to the system.
- If the system state is *θ* < −*θ*_0_ and $\dot{\theta }<-{\dot{\theta }}_{0}$, applying the strongest control effect *f*_4−_ in negative direction to the system.
- If the system state is *θ* < −*θ*_0_ and $|\dot{\theta }|\leq {\dot{\theta }}_{0}$, applying a strong control effect *f*_3−_ in negative direction to the system.
- If the system state is *θ* < −*θ*_0_ and $\dot{\theta }>{\dot{\theta }}_{0}$, applying the weak control effect *f*_2−_ in negative direction to the system.
- If the system state is |*θ*| ≤ *θ*_0_ and $\dot{\theta }<-{\dot{\theta }}_{0}$, applying the weakest control effect *f*_1−_ in negative direction to the system.
- If the system state is |*θ*| ≤ *θ*_0_ and $|\dot{\theta }|\leq {\dot{\theta }}_{0}$, applying the maintaining control effect *f*_0_.

Based on the principle of fuzzy nine-point controller, the fuzzy controller of the rod is shown in Table 1, as well as the fuzzy controller of the cart in Table 2. Note that, in Table 2, the positive direction of the parameter is contrary to Table 1, since the fuzzy controller of the cart is an indirect controller, as is explained by the control logic of the system in Section 2.3.

**Table 1:** Fuzzy controller of the rod.

System state	$\dot{\theta }>{\dot{\theta }}_{0}$	$|\dot{\theta }|\leq {\dot{\theta }}_{0}$	$\dot{\theta }<-{\dot{\theta }}_{0}$
*θ* > *θ*_0_	18	14	10
|*θ*| ≤ *θ*_0_	4	0	-4
*θ* < −*θ*_0_	-10	-14	-18

**Table 2:** Fuzzy controller of the cart.

System state	$\dot{x}>{\dot{x}}_{0}$	$|\dot{x}|\leq {\dot{x}}_{0}$	$\dot{x}<-{\dot{x}}_{0}$
*x* > *x*_0_	-1.9	-1.5	-1.1
|*x*| ≤ *x*_0_	-0.7	0	0.7
*x* < −*x*_0_	1.1	1.5	1.9

According to Figs 1 and 2 and the control strategy mentioned above, for each axis there is only one motor to fulfill the fuzzy control strategy between the rod and the cart. So, a coordinator is needed. In this paper, the coordinator is based on fuzzy inference and evidential reasoning, named the fuzzy-evidential controller, as is shown in detail in the next subsection.

### 3.2 Fuzzy-evidential coordinator

In a close loop feedback control system, the values of control variables are often changing from time to time to keep the dynamic stability of the controlled system, such as the angle of the rod and the displacement of the cart in the planar inverted pendulum system. In this section, the dynamic mass function or dynamic BPA is designed to express the dynamic characteristic of the control variable in the frame of fuzzy inference.

**Fig 4:**
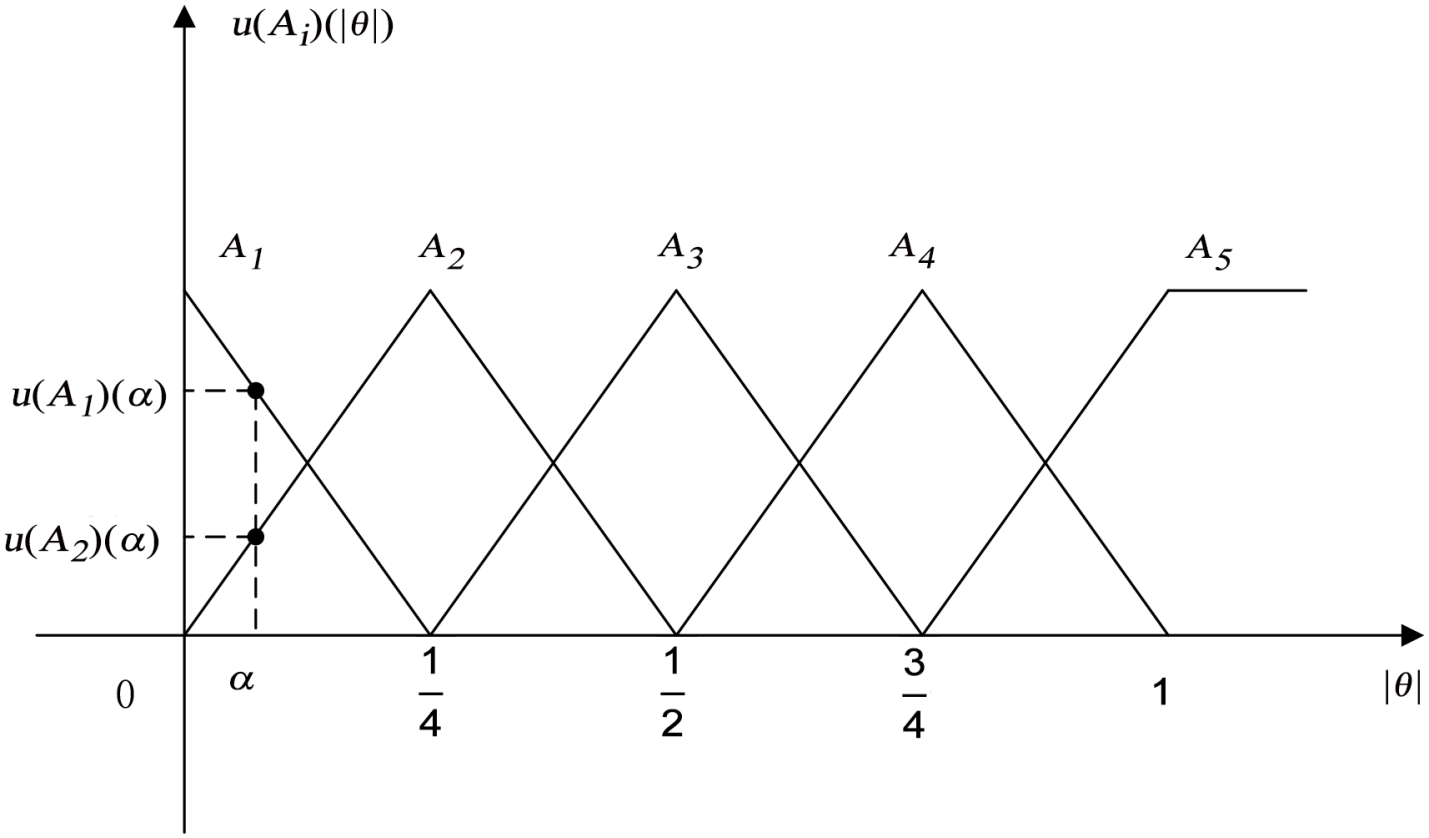
The membership function for constructing BPA.

In Fig 4, Θ = {*A*_1_,*A*_2_,*A*_3_,*A*_4_,*A*_5_) is defined as the frame of discernment with five events, where *A*_*i*_ (*i* = 1,2,3,4,5) is the fuzzy partition of |*θ*| and the value of |*θ*| ranges from a small value to a big one. In this paper, |*θ*| is defined as 0 ≤ |*θ*| ≤ 1. The membership function corresponding to |*θ*| in Fig 4 is defined as *u*(*A*_*i*_) (*i* = 1,2,3,4,5), which satisfies:

$$\sum_{i=1}^{5}u\;(A_{i})=1.$$ (9)

For any given value *θ* in Fig 4, there are at least three zero values among the membership function *u*(*A*_*i*_) (*i* = 1,2,3,4,5), which will lead to a *jump* *value* of a controller in real application, and the discontinuous value in a controller usually has a bad control effect on the controlled object. So the nonzero mass value for each single subset of 2^|Θ|^ is constructed as follows:

$$m\left(A_{i}\right)=k\cdot u\;(A_{i})+BetP\;(A_{i}),\;\left(i=1,2,3,4,5\right),$$ (10)

subject to:

$$\begin{array}{c} 0<k<1,\\ \sum_{i=1}^{5}m(A_{i})=1,\\ BetP\;(A_{i})=\frac{1}{5}\cdot (1-\sum_{i=1}^{5}k\cdot u\;(A_{i})), \end{array}$$ (11)

where *BetP* (*A*_*i*_) is the Pignistic probability for the *i*th fuzzy partition, the mass function *m*(*A*_1_,*A*_2_,*A*_3_,*A*_4_,*A*_5_) is redistributed among all the single subset of 2^|Θ|^ according to *BetP* (*A*_*i*_). The *k* is a scale factor, and a big value of *k* is considered. Since, if the value of *k* is too small, a large value of the originally nonzero membership function in Fig 4 will be redistributed to the fuzzy partition with a zero value, which is contrary to the control logic. According to the control experience, it is recommended to define *k* as a constant parameter and *k* = 0.9. The Eq (11) satisfies Eq (4), which can be derived as follows:

$$\sum_{i=1}^{5}m(A_{i})=\sum_{i=1}^{5}k\cdot u\;(A_{i})+\sum_{i=1}^{5}BetP\;(A_{i})=k\cdot 1+(1-k)=1.$$ (12)

Recall the *weighted* *average* *defuzzifier* as Eq (2) and the *singleton* *fuzzifier* as Eq (1), remember the priority level of the rod is higher than the cart. Mathematically, the coordinator for the fuzzy controller of the rod and the cart is defined as follows:

$$m_{1}=\frac{\sum_{i=1}^{i=5}b_{i}\cdot m\left(A_{i}\right)}{\sum_{i=1}^{i=5}m(A_{i})},m_{2}=\frac{\sum_{i=1}^{i=5}b_{6-i}\cdot m\left(A_{i}\right)}{\sum_{i=1}^{i=5}m(A_{i})},$$ (13)

where *m*_1_ is the weight coefficient for the fuzzy controller of the rod and *m*_2_ is the weight coefficient for the fuzzy controller of the cart. Since *m*(*A*_*i*_) is time-varying because of the dynamic characteristics of the control system, *m*(*A*_*i*_) is a dynamic BPA. The *b*_*i*_ and *b*_6−*i*_ are the equilibrium coefficients to balance the priority level between the fuzzy controller of the rod and the fuzzy controller of the cart. If the deviation of the angle |*θ*| is bigger than the zero zone value *θ*_0_, the fuzzy controller of the rod will work as the master controller which means *b*_*i*_ is a big value and the value of *b*_6−*i*_ will be a small one. Table 3 shows a recommended value of *b*_*i*_ and the corresponding *b*_6−*i*_ (i = 1,2,3,4,5) for the planar inverted pendulum system.

**Table 3:** A recommended value of *b*_*i*_ and *b*_6−*i*_.

*i*	1	2	3	4	5
*b*_*i*_	0.1	0.25	0.5	0.75	0.9
*b*_6−*i*_	0.9	0.75	0.5	0.25	0.1

#### A numerical example

This is an example to show how to calculate the fuzzy-evidential coordinator *m*_1_ and *m*_2_ in Eq (13).

Take the point *α* in Fig 4 as an example. The values of the membership function are *u* (*A*_1_) (*α*) and *u* (*A*_2_) (*α*). It can be proved that *u* (*A*_1_) (*α*) + *u* (*A*_2_) (*α*) = 1, so it satisfies Eq (9). According to Eqs (10) and (11), the dynamic mass function is calculated as follows:

$$\begin{array}{c} m\left(A_{1}\right)\left(\alpha \right)=k\cdot u\left(A_{1}\right)\left(\alpha \right)+\frac{1}{5}\cdot \left[1-k\cdot u\left(A_{1}\right)\left(\alpha \right)-k\cdot u\left(A_{2}\right)\left(\alpha \right)\right],\\ m\left(A_{2}\right)\left(\alpha \right)=k\cdot u\left(A_{2}\right)\left(\alpha \right)+\frac{1}{5}\cdot \left[1-k\cdot u\left(A_{1}\right)\left(\alpha \right)-k\cdot u\left(A_{2}\right)\left(\alpha \right)\right],\\ m\left(A_{3}\right)\left(\alpha \right)=\frac{1}{5}\cdot \left[1-k\cdot u\left(A_{1}\right)\left(\alpha \right)-k\cdot u\left(A_{2}\right)\left(\alpha \right)\right],\\ m\left(A_{4}\right)\left(\alpha \right)=\frac{1}{5}\cdot \left[1-k\cdot u\left(A_{1}\right)\left(\alpha \right)-k\cdot u\left(A_{2}\right)\left(\alpha \right)\right],\\ m\left(A_{5}\right)\left(\alpha \right)=\frac{1}{5}\cdot \left[1-k\cdot u\left(A_{1}\right)\left(\alpha \right)-k\cdot u\left(A_{2}\right)\left(\alpha \right)\right]. \end{array}$$ (14)

Finally, with Eqs (13) and (14), the value of the fuzzy-evidential coordinator for the fuzzy controller of the rod and the cart is calculated as follows:

$$\begin{array}{c} m_{1}\;(\alpha )=\frac{\sum_{i=1}^{i=5}b_{i}\cdot m(A_{i})\;(\alpha )}{\sum_{i=1}^{i=5}m(A_{i})\;(\alpha )},m_{2}\;(\alpha )=\frac{\sum_{i=1}^{i=5}b_{6-i}\cdot m(A_{i})\;(\alpha )}{\sum_{i=1}^{i=5}m(A_{i})\;(\alpha )}. \end{array}$$ (15)

## 4 Experiment

The stability of the planar inverted pendulum system in the sense of Lyapunov is proved in [6]. The purpose of this experiment is to verify the effectiveness of the proposed fuzzy-evidential controller. The experiment is realized in MATLAB, the nonlinear model of the planar inverted pendulum in reference [8] is chosen to be the model of the controlled system in simulation platform. Each fuzzy evidential controller, as well as the planar inverted pendulum is expressed as a *S*-function in MATLAB.

**Fig 5:**
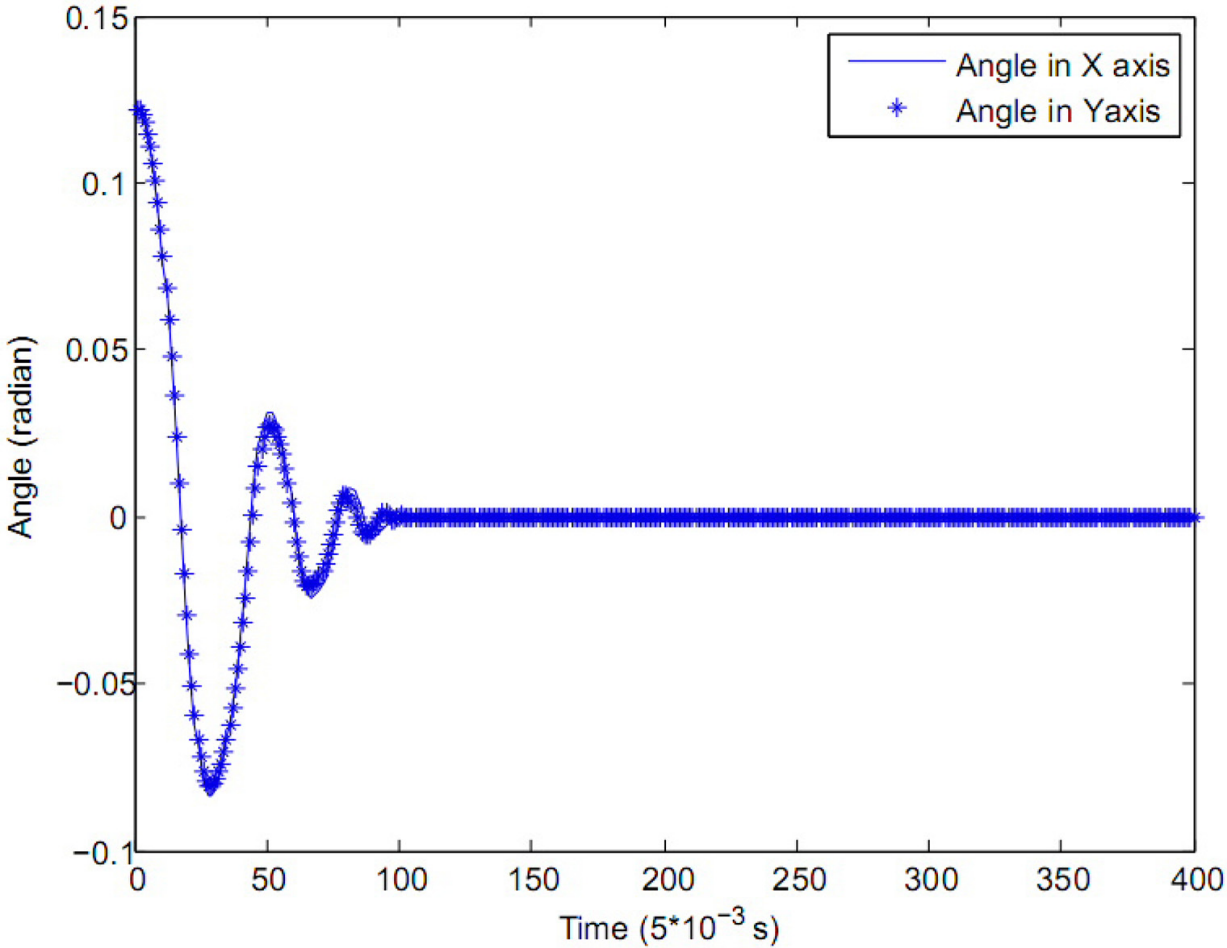
The deviation of the rod in each axis.

**Fig 6:**
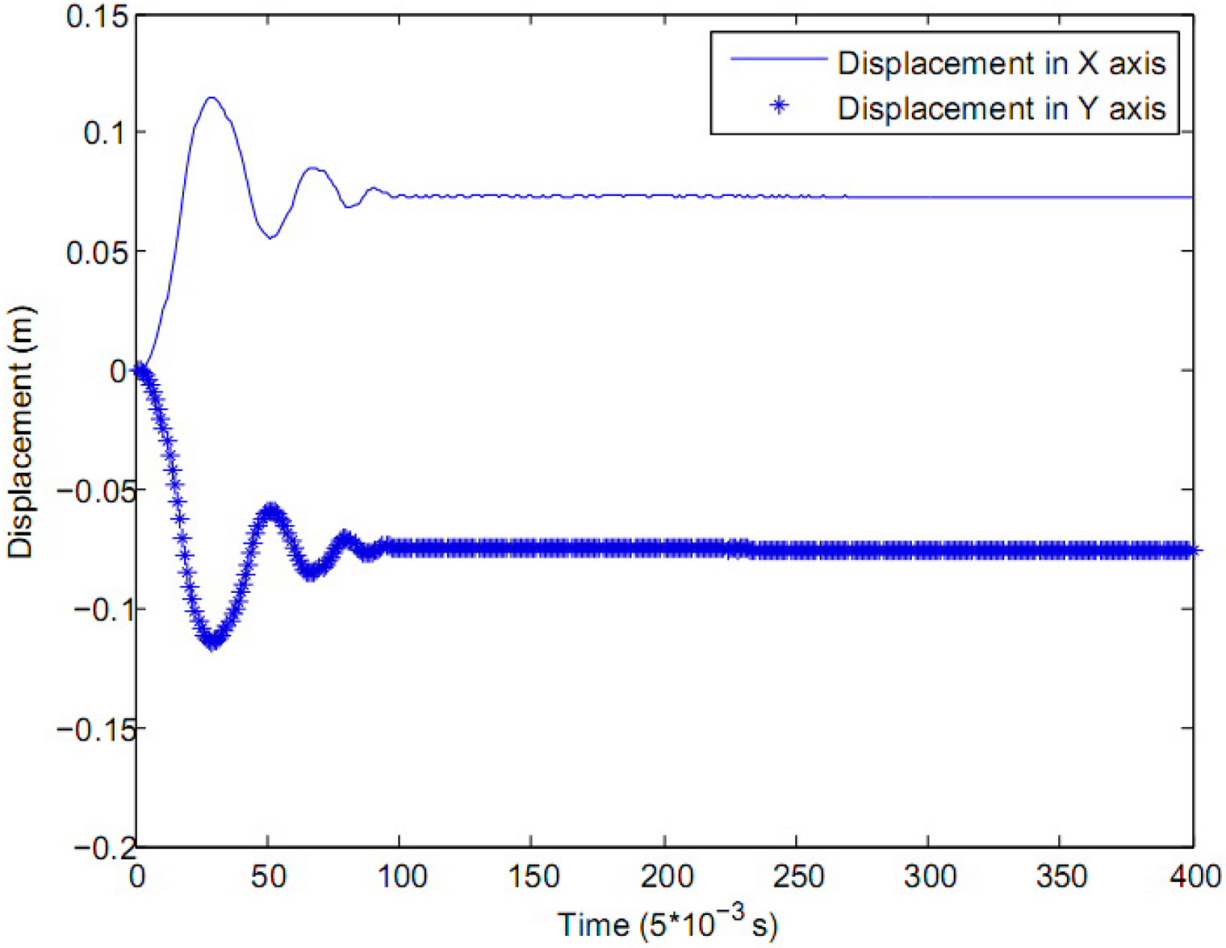
The displacement of the cart in each axis.

**Fig 7:**
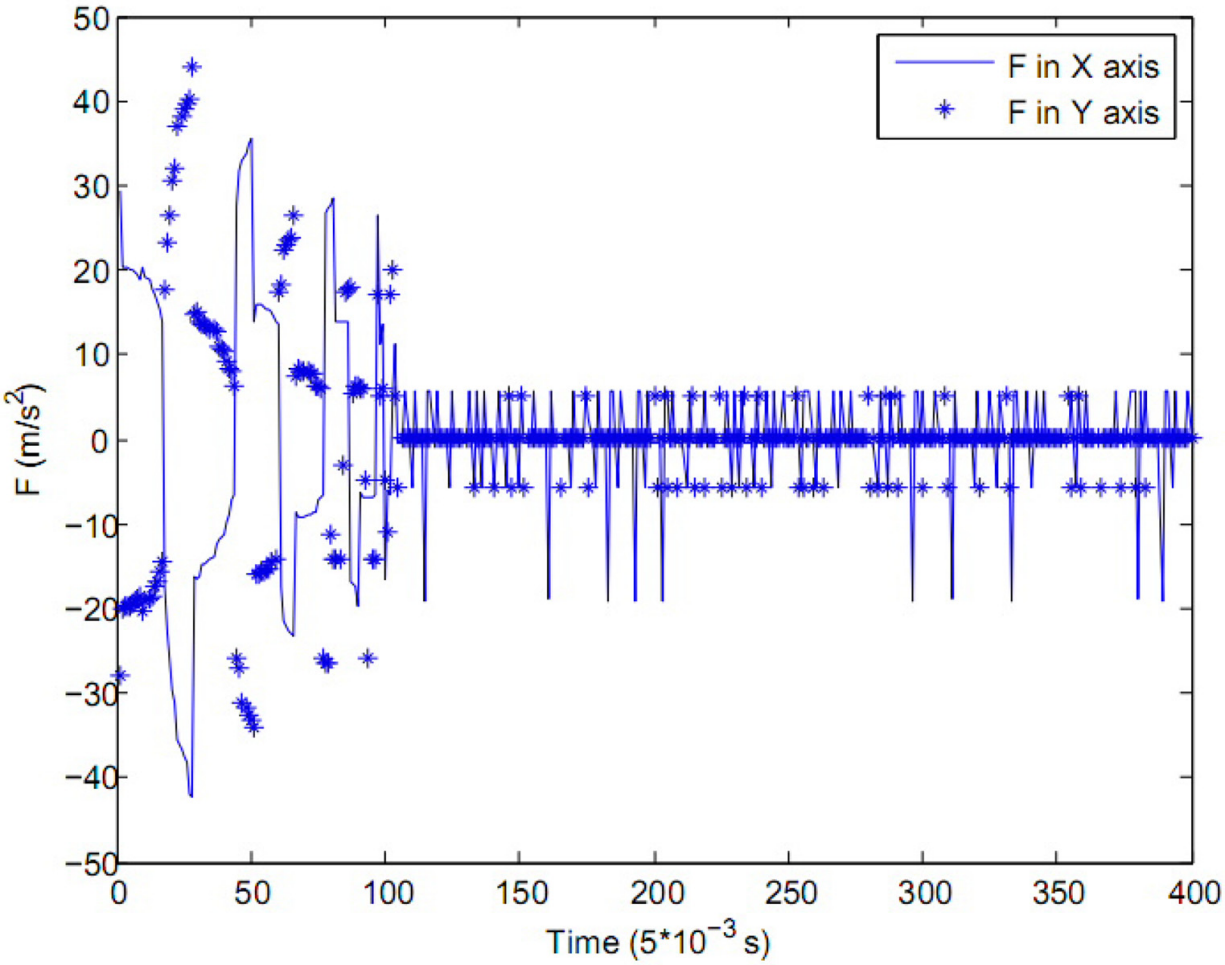
The control effect for each axis.

The results of the control experiment are shown in Figs 5, 6 and 7, where the solid line represents the control variable in *X*-axis and the star line represents the control variable in *Y*-axis, the control cycle of the close loop is 0.005 *second*.

In Fig 5, both in *X*-axis and *Y*-axis, the initial deviation of the rod from its upright position is 7 *degree*, which is about 0.122 *radian*. The zero zone value of the fuzzy controller for the rod in Table 1 is *θ*_0_ = 0.007 *rad* and ${\dot{\theta }}_{0}=0.009$ *rad*/*s* in both *X*-axis and *Y*-axis. According to Fig 6, the zero zone value of the fuzzy controller for the rod in Table 1 is *x*_0_ = 0.018 *m* and ${\dot{x}}_{0}=0.04$ *m*/*s* in both *X*-axis and *Y*-axis. After about 0.5 *second*, the displacement of the cart becomes stable, and keep adjustment near the zero point. The control variables in Figs 5 and 6 will go to a stable state in about 0.5 *second*, which means the stabilization of the controlled system. Compared with the control effect in reference, the method in this paper has a better control effect than the LQR method [8] and the fuzzy controller [12], the comparative result among these three methods is shown in Table 4. The overshoot of the angle and displacement in the proposed method is smaller than the LQR method in [8], so the adjusting time of the proposed method is shorter than LQR. Also, there is less zero value in the proposed method, and the control variable is smoother than the classical fuzzy controller in [12], which means the fuzzy-evidential coordinator is more effective, so the time to the stabilization needed by the controlled system decreases obviously.

**Table 4:** Time before the stabilization of the system.

Control method	time(s)
LQR controller [8]	3
Fuzzy controller [12]	1.2
Fuzzy-evidential controller	0.5

Fig 7 shows the control effect in each axis, which is an acceleration control variable comes from the motor of each axis. In Fig 7, the control effect in each axis is a big value at first, then it decreases to a small value and maintain fine tuning, this is in harmony with Figs 5 and 6. The final control effect for *X*-axis ranges from [-45,35] and [-35,45] for *Y*-axis.

The data of the experiment results can be found in Supporting Information.

## 5 Conclusion

This paper proposes a new fuzzy-evidential controller for the stabilization of the planar inverted pendulum system. After designing a fuzzy controller for the rod and the cart, a fuzzy-evidential coordinator for these two controllers is proposed based on fuzzy inference and evidential reasoning. The experimental result shows the effectiveness of the proposed method, as well as the merit that the new controller can force the system into its stable state faster than the method without evidential reasoning or based on LQR in the references. The following work includes extending this method to other application areas, as well as the more complicated inverted pendulum, i.e. the double and triple planar inverted pendulum.

## Supporting Information

S1 Table The data of the experiment results. (XLSX)

S1 File The data file. (TXT)
